# Edaravone dexborneol protected neurological function by targeting NRF2/ARE and NF-κB/AIM2 pathways in cerebral ischemia/reperfusion injury

**DOI:** 10.3389/fphar.2025.1581320

**Published:** 2025-04-25

**Authors:** Hui Zhang, Can Zhu, Xingyu Zhou, Laifa Wang, Ling Deng, Binsheng He, Jianming Li

**Affiliations:** ^1^ Hunan Provincial University Key Laboratory of the Fundamental and Clinical Research on Neurodegenerative Diseases, Changsha Medical University, Changsha, China; ^2^ The Hunan Provincial University Key Laboratory of the Fundamental and Clinical Research on Functional Nucleic Acid, Changsha Medical University, Changsha, China; ^3^ Hunan Provincial Key Laboratory of the Research and Development of Novel Pharmaceutical Preparations, Changsha Medical University, Changsha, China; ^4^ Hunan Provincial Key Laboratory of the TCM Agricultural Biogenomics, Changsha Medical University, Changsha, China

**Keywords:** edaravone dexborneol, neuroprotection, Nrf2/ARE pathway, NF-κB/AIM2 pathway, cerebral ischemia/reperfusion injury

## Abstract

**Background:**

Edaravone dexborneol (Eda-Dex), a promising neuroprotectant composed of edaravone and (+)-borneol, has been clinically applied in stroke treatment. However, the mechanism of action of Eda-Dex remains unclear.

**Methods:**

A rat model of cerebral ischemia/reperfusion injury (CIRI) was created through middle cerebral artery occlusion. Neurological scoring, TTC staining, and laser speckle imaging were used to assess neurological deficits, infarct size and cerebral blood flow (CBF). Behavioral tests, including the open field test, the elevated plus maze, and the novel object recognition test, were conducted to assess animal behavior. Western blotting and ELISA were employed to assess levels of expression of components of the NRF2/ARE and NF-κB/AIM2 pathways and of specific cytokines. The levels of oxidative stress markers were analyzed via commercially available kits. HE staining, Nissl staining, and immunohistochemistry were used to assess pathological alterations in the brain.

**Results:**

Eda-Dex dramatically reduced the neurological deficit score and cerebral infarct size, increased CBF, and attenuated anxiety-like behavior and improved cognitive function in CIRI rats. Eda-Dex significantly reduced oxidative stress and relieved inflammatory response and it significantly upregulated NRF2, NQO1, HO-1, and SLC7A11 and significantly downregulated NF-κB, AIM2, ASC and caspase 1 in the infarcted brain. Moreover, Eda-Dex clearly reduced pathological damage, rescued neurons, and reduced the activation of microglia and astrocytes.

**Conclusions:**

The results of this study confirm that Eda-Dex exerts neuroprotective effects by synergistically inhibiting oxidative stress and inflammation via the NRF2/ARE and NF-κB/AIM2 pathways in CIRI rats.

## 1 Introduction

Stroke is currently the second most common cause of death globally and is known for its high incidence, high rate of causing disability, and high fatality rate. Ischemic stroke is the primary type of stroke, accounting for approximate 80% of all stroke cases (Hilkens et al., 2024; Liang et al., 2023). Clinically, the most widely applied treatments for ischemic stroke are vascular recanalization-based strategies, such as reperfusion with recombinant tissue plasminogen activator (rtPA). However, these therapies are limited by potential bleeding risk and narrow time windows for application, and thrombolytic drugs cannot reverse long-term motor and cognitive dysfunction after ischemic stroke (Herpich and Rincon, 2020; Yao et al., 2023). Additionally, studies have shown that swift re-establishment of blood flow following ischemia leads to significant brain impairment known as cerebral ischemia/reperfusion injury (CIRI) (Lee et al., 2017). This condition, which results from a complex interplay among inflammation, oxidative stress, calcium overload, excitatory amino acid toxicity, mitochondrial dysfunction, apoptosis, autophagy, and ferroptosis, highlights the need for multifaceted treatment approaches that target the various pathways involved in CIRI (Lou et al., 2018). Stroke patients usually suffer from serious physical and cognitive impairment. Poststroke cognitive impairment, the prevalence of which ranges from 20% to 80% among stroke patients, is more likely to be ignored than severe physical disability (Hugues et al., 2021).

Edaravone, approved in Japan in 2001, acts as a free radical scavenger and is used in the treatment of acute ischemic stroke (Ishihara and Suzuki, 2016). (+)-Borneol is a component of D-borneolum, which has anti-inflammatory activity and has long been used in China to relieve injury due to ischemic stroke (Dong et al., 2018). Eda-Dex, a new neuroprotective compound that combines edaravone and (+)-borneol in a 4:1 M ratio, was granted approval for use in China in 2020. It has been shown to decrease cerebral nerve damage and expand the treatment window efficiently (Fu R. et al., 2024). It has been hypothesized that Eda-Dex has synergistic antioxidative and anti-inflammatory effects and that it is a potent and cost-effective agent for treating stroke (Wu et al., 2014; Xu et al., 2021).

Nrf2 is a central regulator of the cellular antioxidant defense system and plays an important role in the pathogenesis of cerebral ischemic injury. During oxidative stress, Nrf2 moves into the nucleus, where it forms complexes with antioxidant response elements (AREs) and triggers the activation of target genes such as HO-1, GPX4, NQO1, and SLC7A11 (Dong et al., 2020; Luo et al., 2023). The NF-κB pathway plays a broad regulatory role in pathophysiological processes and is a promising therapeutic target for the treatment of stroke (Wang et al., 2017). Not only can NF-κB activate the expression of proinflammatory and antiapoptotic genes, it can also participate in various types of signal crosstalk during inflammasome activation (Bao et al., 2016; Yi J et al., 2023). The AIM2 inflammasome is an essential mediator of neuroinflammation and provides a valuable therapeutic target for ischemic stroke (Fu Y. et al., 2024). On the basis of the above evidence, we hypothesized that Eda-Dex protects neurological function in CIRI rats by synergistically inhibiting oxidative stress and inflammation through targeting of the NRF2/ARE and NF-κB/AIM2 pathways.

## 2 Materials and methods

### 2.1 CIRI model

**FIGURE 1:**
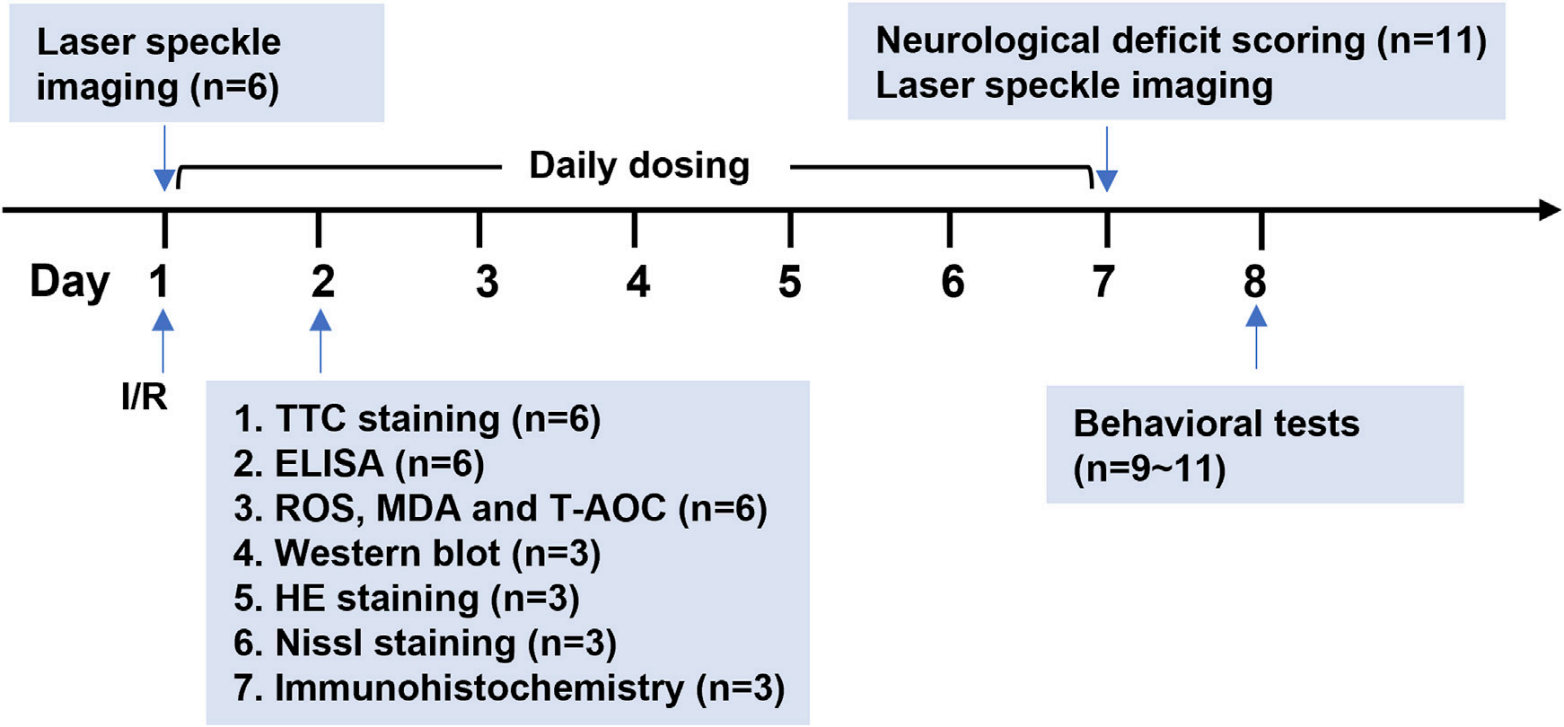
Procedure used in the animal experiment. CIRI model rats were established by MCAO surgery. CBF was monitored before MCAO, immediately after MCAO and after reperfusion and on the 7th day after MCAO. Eda-Dex (4 mL/kg) was administered to the model rats once daily for 7 consecutive days. Infarct area, inflammatory factors, oxidative stress factors, expression of relevant signaling pathways, and histopathological changes were analyzed 24 h after MCAO. Neurological function was evaluated on the 7th day. Animal behavioral tests were started on the 8th day.

All animal procedures used in this study were approved by the Animal Care and Use Committee of Changsha Medical University. SD rats (male, 250–280 g) were purchased from Hunan SJA Laboratory (Changsha, China). The animals were randomly divided into three groups: a sham group, a Veh + CIRI group and an Eda-Dex + CIRI group. The CIRI model was created via middle cerebral artery occlusion (MCAO) following Longa’s technique (Longa et al., 1989). Briefly, the rats were anesthetized with pentobarbital sodium (30 mg/kg, i.p.), and the right middle cerebral artery of each rat was occluded with a nylon suture (Cinontech, Beijing, China). After a 2-h period of occlusion, the suture was removed to allow reperfusion. The CIRI rats were intraperitoneally administered 4 mL/kg Eda-Dex (2 mg/mL eladavone and 0.5 mg/mL (+)-borneol; Simcere Pharmaceutical Group, Nanjing, China) once a day for 7 consecutive days. The dosages of Eda-Dex used were determined based on drug specifications and the results of previous studies (Hua et al., 2015). A detailed flowchart of the animal experiment is presented in Figure 1.

### 2.2 Neurological deficit scoring

Neurological impairment was evaluated according to the following criteria: 0, normal; 1, weak forelimbs; 2, turning in a circle to the diseased side; 3, inability of the affected side to bear weight; and 4, inability to move (Shah et al., 2006). Rats that received scores of 0 or 4 were excluded from the subsequent experiments.

### 2.3 Laser speckle imaging

Cerebral blood flow (CBF) was analyzed using a laser speckle imaging system (RWD Life Technologies, Shenzhen, China). The relative CBF in the region of interest (ROI) is presented as the ratio of the CBF in the right hemisphere to that in the left hemisphere.

### 2.4 2,3,5-Triphenyltetrazolium chloride (TTC) staining

Briefly, fresh brains were frozen at −20°C for 20 min, and each brain was cut into 5 slices with a thickness of 2 mm. The slices were stained with 1% TTC (Solarbio Life Sciences, Beijing, China) solution at 37°C for 30 min and fixed in 4% paraformaldehyde at 4°C overnight. The infarct size was determined using ImageJ (Media Cybernetics, Bethesda, MD, United States).

### 2.5 Open field test (OFT)

Following a 2-hour acclimation period, the rats were gently placed in the central area of the open field and allowed 5 min of unrestricted movement. The travel distance and the time spent in the central area were recorded via Smart 3.0 (Panlab, Spain) (Zhang et al., 2023a).

### 2.6 Elevated plus maze (EPM)

Following a 2-hour acclimation period, the rats were gently positioned in the central area facing the open arm and allowed 5 min of unrestricted exploration. The amounts of time spent in the open and closed arms of the maze were recorded (Zhang H. et al., 2024).

### 2.7 Novel object recognition (NOR) test

In the adapting phase, the rat was placed in the testing box, which contained two identical objects, and allowed to move freely for 5 min. At the start of the testing phase, the rat was removed from the box, and one of the existing objects was replaced by a novel object. The rat was then returned to the box and allowed to explore freely for 10 min. The time the animal spent sniffing or climbing each object was recorded. The discrimination ratio was calculated according to the following formula: (Time _Novel_-Time _Familiar_)/(Time _Novel_+Time _Familiar_) (Jaiswal et al., 2019).

### 2.8 Enzyme-linked immunosorbent assay (ELISA)

The levels of IL-6 (product number: MM-0190R1), IL-1β (product number: MM-0047R1) and TNF-α (product number: MM-0180R1) in the ischemic cortex were measured using Meiman^®^ ELISA kits (Jiangsu, China). The standards were prepared by gradient dilution of the original density standard. A total of 50 μL of standard was added to the wells. A total of 2 μL of supernatants of cortex homogenate was mixed 48 μL of sample diluent and added to the wells. The plate was incubated at 37°C for 30 min followed by washing 5 times. A total of 50 μL HRP-conjugate reagent was added to each well except for the blank well. The plate was incubated at 37 °C for 30 min followed by washing 5 times. Chromogen Solution A (50 μL) and Chromogen Solution B (50 μL) were sequentially added to each well. The plate was incubated at 37°C for 15 min. The reaction was stopped by adding 50 μL stop solution to each well. The absorbance was measured at 450 nm. The concentrations (pg/mg prot) of individual cytokines were calculated based on standard curves.

### 2.9 Reactive oxygen species (ROS) analysis

ROS were detected via an ROS assay kit (Jiancheng Bioengineering Institute, Nanjing, China) according to the manufacturer’s instructions. The absorbance was measured at an excitation wavelength of 500 nm and an emission wavelength of 525 nm. The ROS level is reported as the fold change in the absorbance value relative to the value obtained for the sham group.

### 2.10 Malondialdehyde (MDA) analysis

MDA levels were measured via an MDA assay kit (Jiancheng Bioengineering Institute, Nanjing, China) according to the manufacturer’s instructions. The optical density (OD) at 532 nm was measured. MDA (nM/mgprot) = OD _Sample_×10 nM/(OD _Standard_–OD _Blank_)/protein concentration.

### 2.11 Total antioxidant capacity (T-AOC) analysis

T-AOC was measured using a T-AOC assay kit (Sangon Biotech, Shanghai, China). The OD at 515 nm was measured according to the manufacturer’s instructions. T-AOC (μM/mg prot) = 1.414×(OD _Blank_–OD _Sample_+0.0081)/protein concentration.

### 2.12 Western blotting

Protein was isolated from ischemic cortex and measured via the BCA method. The samples were separated via SDS‒PAGE and transferred to nitrocellulose membranes. The membranes were subsequently blocked with 5% nonfat milk for 1 h and incubated with primary antibodies at 4°C overnight. The primary antibodies used included anti-NRF2 (1:500, D121053, Sangon Biotech, Shanghai, China), anti-HO-1 (1:200, D220756, Sangon Biotech, Shanghai, China), anti-NQO1 (1:500, D161049-0025, Sangon Biotech, Shanghai, China). anti-SLC7A11 (1:500, D262619, Sangon Biotech, Shanghai, China), anti-GAPDH (1:1000, D110016, Sangon Biotech, Shanghai, China), anti-AIM2 (1:1000, 20590-1-AP, ProteinTech Group, Chicago, United States), anti-ASC (1:1000, 30641-1-AP, ProteinTech Group, Chicago, United States), anti-caspase 1/p20/p10 (1:500, 22915-1-AP, ProteinTech Group, Chicago, United States). After being washed 3 times with TBST, the membranes were incubated with Alexa Fluor 680-conjugated donkey anti-rabbit IgG (1:10000, D110016, Sangon Biotech, Shanghai, China) and washed again. The bands were visualized on an Odyssey CLx system (Li-COR, United States), quantified using ImageJ and normalized to GAPDH.

### 2.13 HE staining, Nissl staining and immunohistochemistry

The brains were fixed in 4% paraformaldehyde overnight, sequentially dehydrated in 15% sucrose, 30% sucrose and 35% sucrose (12 h each), and embedded in Tissue-Tek^®^ O.C.T. (Sakura Finetek, United States) for frozen sectioning. The morphology and structure of the lesions were examined via HE staining (Sangon Biotech, Shanghai, China). The pathological changes were observed under a microscope, and the percentage of the total area that showed pathology was assessed [29]. Nissl staining was performed using a Nissl staining kit (Solarbio, Beijing, China). The number of Nissl bodies was then calculated. The primary antibodies used in immunohistochemistry were an anti-GFAP polyclonal antibody (1:200, ab7260, Abcam, United States) and anti-Iba-1 antibody (1:200, 019-19741, Wako, Japan). Five sections were randomly chosen, and the number of positive cells per field was determined.

### 2.14 Statistical analysis

Statistical analysis was performed by GraphPad Prism software version 8.0.2 (San Diego, CA, United States). Normality of the data was tested by the Shapiro-Wilk test. Homogeneity of variance was tested by the Bartlett’s test. The data were expressed as the means±SEMs. Multiple comparisons were analyzed by one-way ANOVA with Tukey’s *post hoc* test. Comparison between the two groups was analyzed by unpaired Student’s t-test. *p* < 0.05 was considered to indicate statistical significance.

## 3 Results

### 3.1 Eda-Dex lowered neurological scores, minimized cerebral infarct size, and enhanced CBF in CIRI rats

**FIGURE 2:**
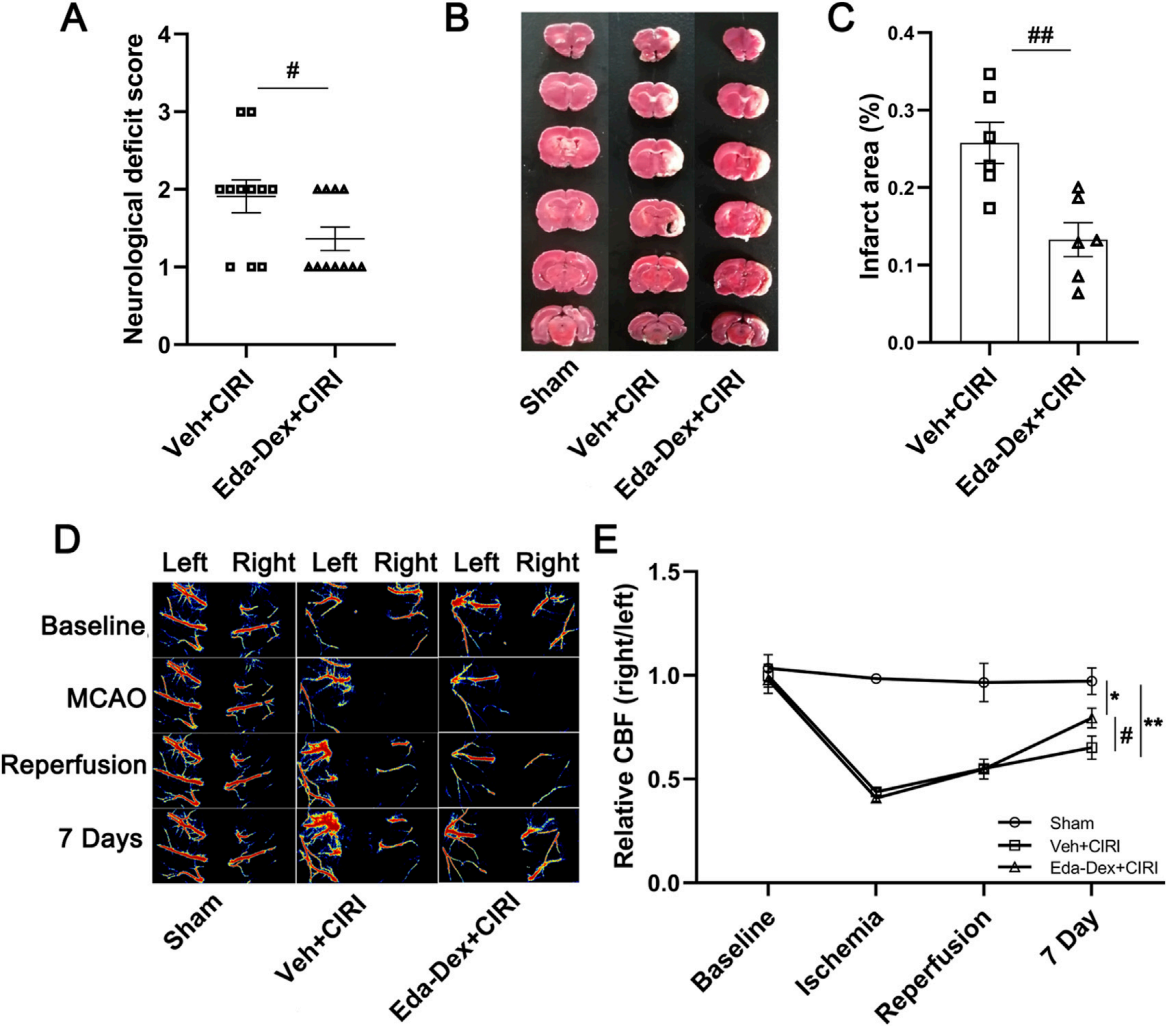
Influences of Eda-Dex on neurological deficits, CBF, and infarct area. **(A)** Neurological function scoring. CIRI resulted in obvious neurological deficits, whereas Eda-Dex significantly improved neurological function. The data are presented as the means±SEMs and were analyzed by unpaired Student’s t-test (n = 11). **(B, C)** Infarct area was analyzed by TTC staining. CIRI resulted in obvious infarction in the right hemisphere of the brain, whereas Eda-Dex significantly reduced the infarct area. The data are presented as the means±SEMs and were analyzed by unpaired Student’s t-test (n = 6). **(D, E)** CBF was monitored by laser speckle imaging. The CBF in the right hemisphere of the brain obviously decreased after MCAO. The CBF was significantly improved on the 7^th^ day. The data are presented as the means±SEMs and were analyzed by one-way ANOVA followed by Tukey’s *post hoc* test (n = 6). *p < 0.05, and **p < 0.01 versus the sham group; ^#^ *p* < 0.05, and ^##^ *p* < 0.01 versus the veh + CIRI group.

Neurological function was assessed on the 7th day following CIRI. The rats in the sham group behaved normal without any obvious neurological impairment (data not shown). The neurological scores of the animals in the CIRI group were ranging from 1 to 3 and were significantly reduced by Eda-Dex (*p =* 0.049) (Figure 2A). According to the TTC results, normal rats showed no obvious infarct area. Notable infarct area was induced by CIRI, whereas the infarct area was significantly reduced by Eda-Dex (*p =* 0.047) (Figures 2B, C). Laser speckle imaging revealed that the CBF in the right hemisphere of the brain obviously decreased after MCAO. After administration of Eda-Dex for 7 consecutive days, the CBF of the animals in the Eda-Dex+CIRI group was significantly greater than that of the animals in the CIRI group (*F =* 24.74, *p* = 0.046) (Figures 2D, E).

### 3.2 Eda-Dex attenuated anxiety-like behavior and cognitive impairment in CIRI rats

**FIGURE 3:**
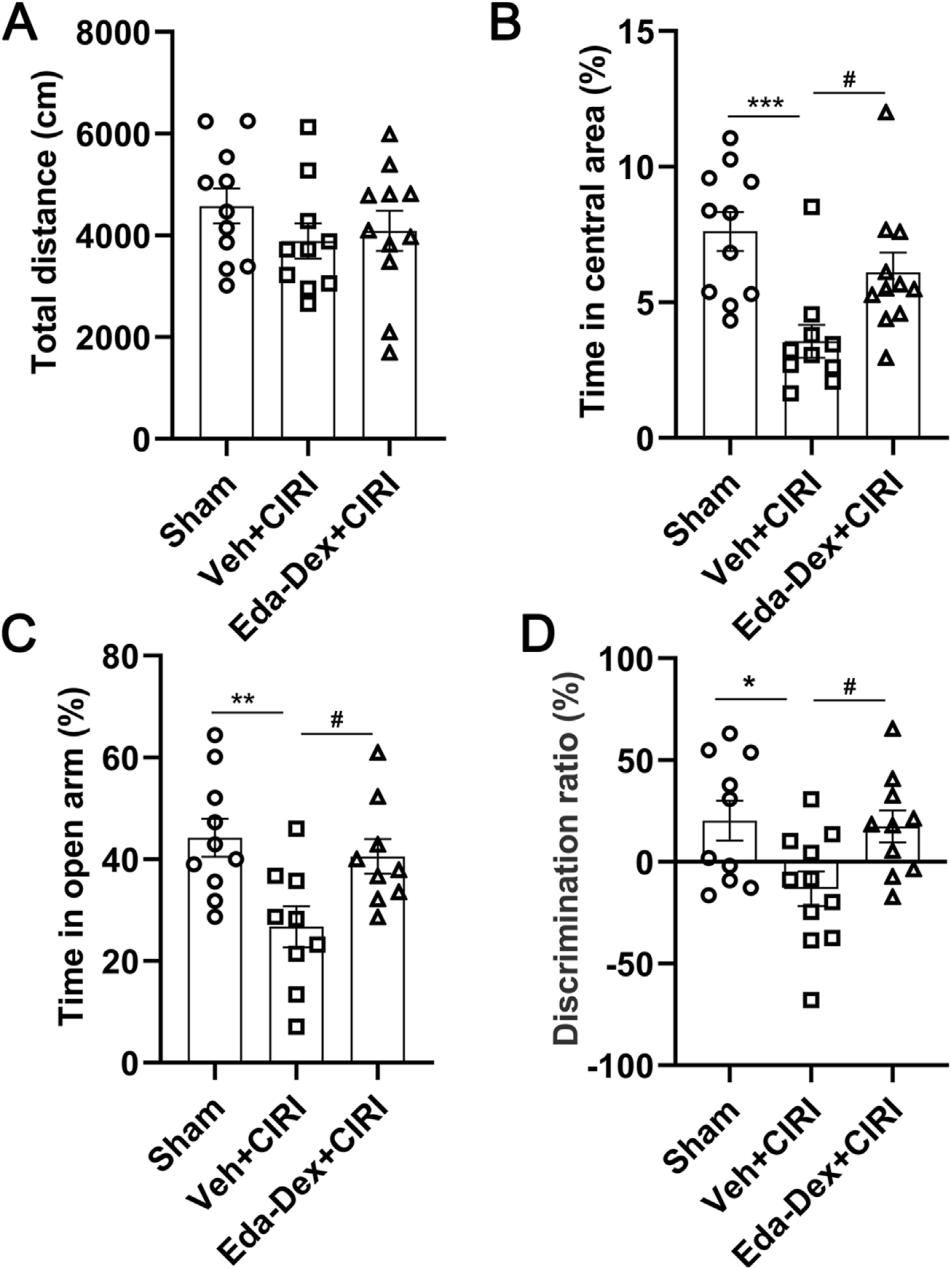
Influences of Eda-Dex on animal behavior. **(A)** Travelling distance was analyzed by OFT. There no obvious differences in travelling distance among the three groups. Anxiety-like behavior was analyzed by **(B)** OFT and **(C)**EPM. CIRI resulted in a significant reduction in the time spent in the central area or the open arms, whereas Eda-Dex significantly inhibited this increase. **(D)** Learning and memory ability was analyzed via the NOR test. The discrimination ratio was significantly decreased by CIRI and significantly increased after Eda-Dex treatment. The data are presented as the means±SEMs and were analyzed by one-way ANOVA followed by Tukey’s *post hoc* test (n = 10–11). *p < 0.05, **p < 0.01, and ***p < 0.001 versus the sham group; ^#^ *p* < 0.05 versus the veh + CIRI group.

The OFT results revealed no notable differences in travel distance among the three groups, suggesting that behavioral dysfunction was attributed to CIRI rather than locomotion alterations (Figure 3A). Compared with the sham group, the CIRI group presented a significant reduction in time spent in the central area (*p* = 0.0008), whereas administration of Eda-Dex significantly inhibited this decrease (*F =* 8.65, *p* = 0.038) (Figure 3B). EPM revealed that CIRI rats spent significantly less time in the open arms of the maze than did the rats in the sham group (*p* = 0.0073) and that the time spent in the open arms by the rats that received Eda-Dex was significantly different (*F =* 6.06, *p* = 0.042) (Figure 3C). The NOR test revealed that the CIRI group presented a significant decrease in discrimination ratio (*p* = 0.028), and this was reversed by administration of Eda-Dex (*F =* 4.65, *p* = 0.048) (Figure 3D). The data above suggest that Eda-Dex effectively alleviates anxiety-like behavior and enhances learning and memory capability in CIRI rats.

### 3.3 Eda-Dex decreased the levels of inflammatory cytokines in CIRI rats

**FIGURE 4:**
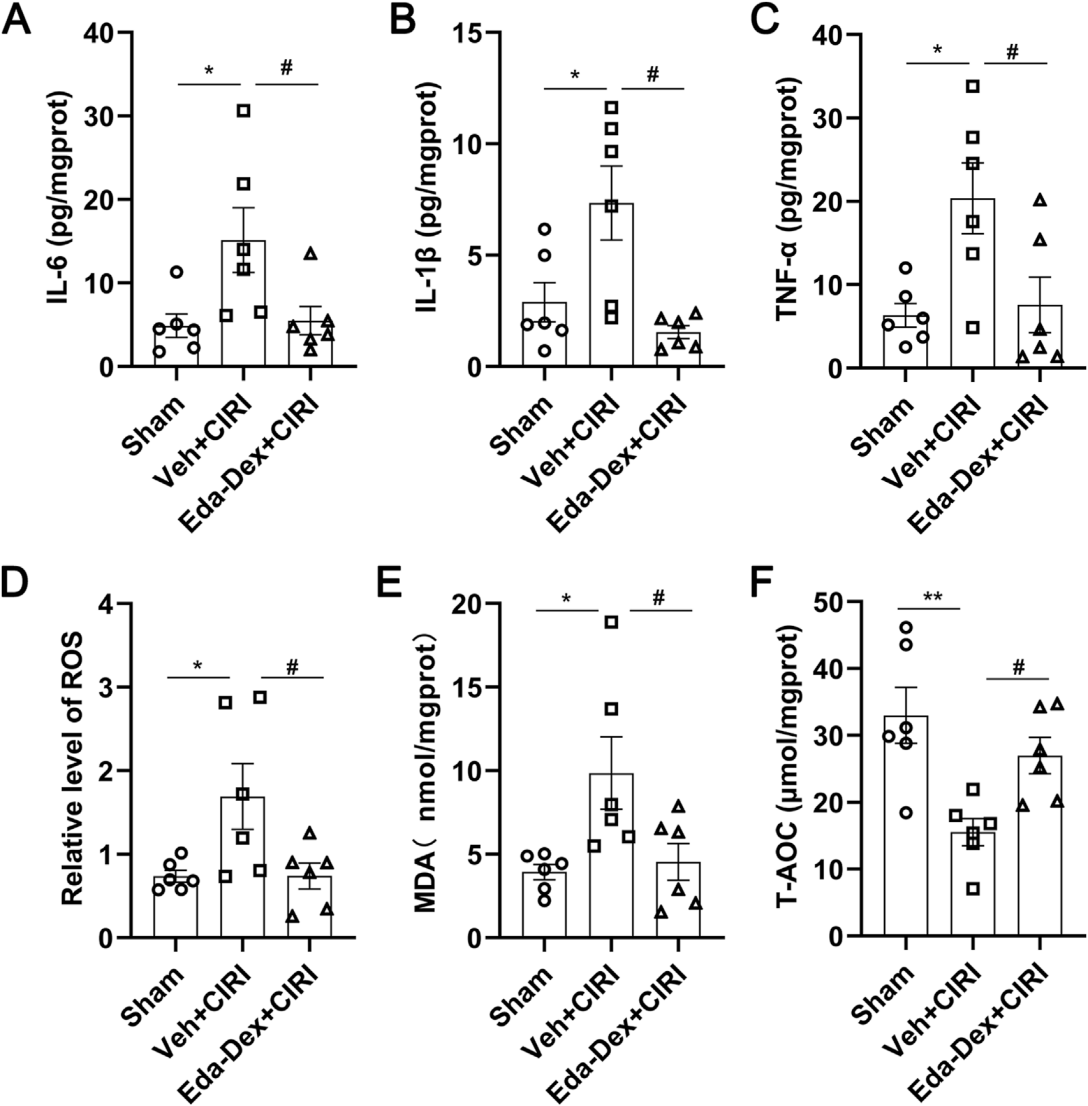
Influences of Eda-Dex on inflammation and oxidative stress. **(A–C)** Influences of Eda-Dex on inflammatory factors, including IL-6, IL-1β, and TNF-α. The levels of the inflammatory factor were significantly increased in response to CIRI and significantly reduced after Eda-Dex treatment. **(D–F)** Influences of Eda-Dex on oxidative stress associated factors, including ROS, MDA, and T-AOC. The levels of ROS and MDA were significantly increased and the level of T-AOC was significantly decreased in response to CIRI, whereas Eda-Dex reversed the effects of CIRI. The data are presented as the means±SEMs and were analyzed by one-way ANOVA followed by Tukey’s *post hoc* test (n = 6). *p < 0.05, and **p < 0.01 versus the sham group; ^#^ *p* < 0.05, and ^##^ *p* < 0.01 versus the veh + CIRI group.

The levels of IL-6 (Figure 4A), IL-1β (Figure 4B) and TNF-α (Figure 4C) in the supernatants of the ischemic cortex homogenate were markedly elevated in CIRI rats compared with those in the sham group (*p* = 0.033; *p* = 0.030; *p* = 0.002). Treatment with Eda-Dex significantly suppressed this increase (*F =* 4.97, *p* = 0.046; *F =* 7.62, *p* = 0.0054; *F =* 5.81, *p* = 0.034).

### 3.4 Eda-Dex decreased the levels of ROS and MDA, and increased the level of T-AOC in CIRI rats

ROS (Figure 4D) and MDA (Figure 4E) levels in the supernatants of the ischemic cortex homogenate were significantly greater in the animals in the CIRI group than in those in the sham group (*p* = 0.038; *p* = 0.026), and this increase was significantly suppressed by administration of Eda-Dex (F = 4.98, *p* = 0.039; F = 5.22, *p* = 0.047). T-AOC activity was markedly lower in the CIRI group than in the sham group (*p* = 0.0033), and this decrease was effectively prevented by administration of Eda-Dex (*F =* 8.16, *p* = 0.049) (Figure 4F). These data suggest that Eda-Dex potently inhibits the overactivation of oxidative stress that is induced by CIRI.

### 3.5 Eda-Dex activated NRF2/ARE signaling in CIRI rats

**FIGURE 5:**
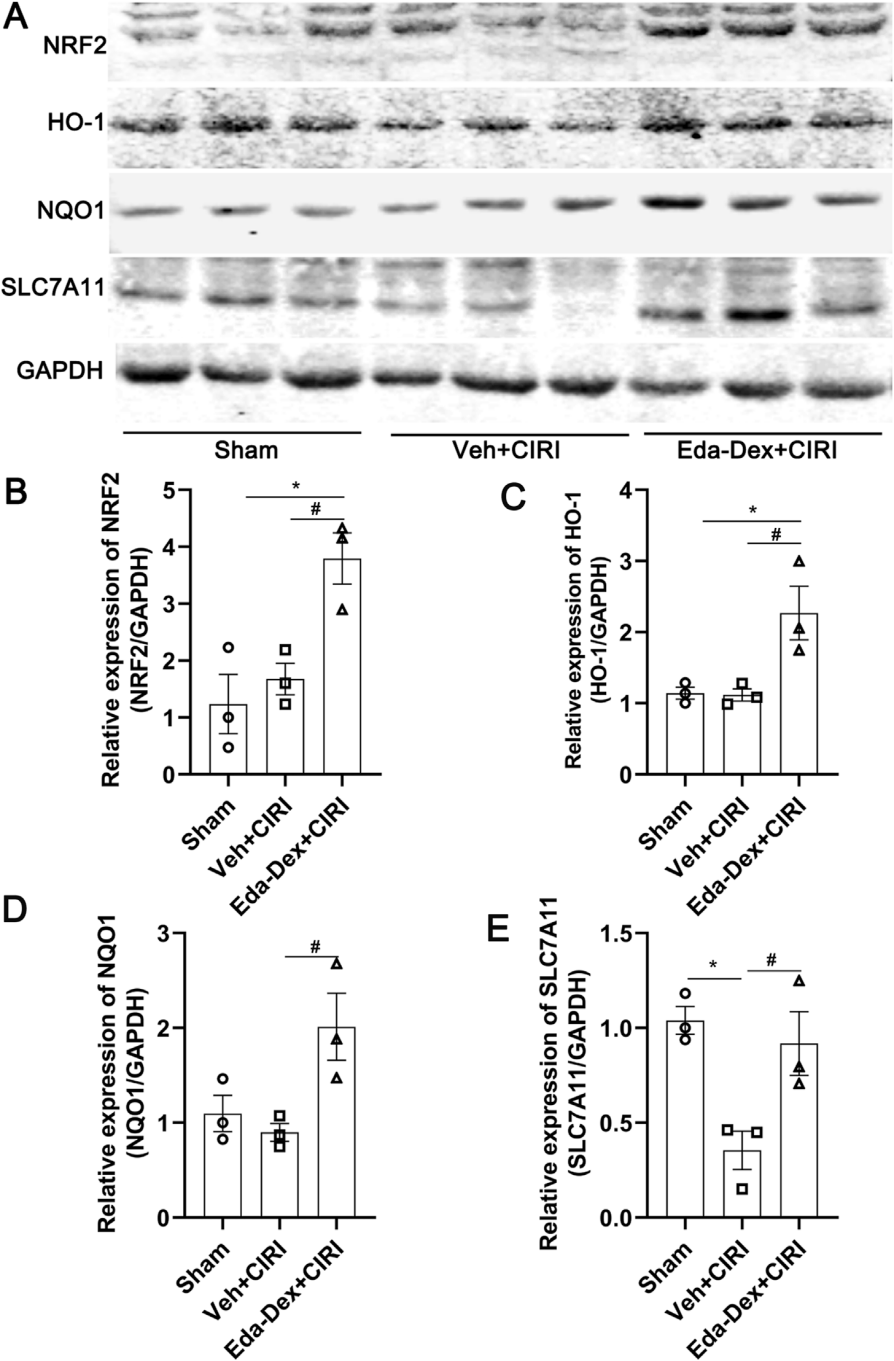
Influences of Eda-Dex on the NRF2/ARE signaling pathway. **(A)** Representative images of Western blotting and quantification of the expression levels of **(B)** NRF2, **(C)** HO-1, **(D)** NQO1, and **(E)** SLC7A11. Eda-Dex significantly increased the expression levels of these proteins in CIRI rats. The data are presented as the means±SEMs and were analyzed by one-way ANOVA followed by Tukey’s *post hoc* test (n = 3). *p < 0.05 versus the sham group; ^#^ *p* < 0.05 versus the veh + CIRI group.

The expression of NRF2/ARE signaling in the ischemic cortex was analyzed by Western blotting. As shown in Figure 5, NRF2 levels increased substantially in the animals that received Eda-Dex treatment (*F* = 10.20, *p* = 0.03). The levels of HO-1, NQO1 and SLC7A11 were also significantly increased after Eda-Dex administration (*F* = 8.31, *p* = 0.027; *F* = 6.20, *p* = 0.038; *F* = 9.12, *p* = 0.038). These results suggest that Eda-Dex inhibits oxidative stress by targeting the NRF2/ARE pathway.

### 3.6 Eda-Dex suppressed the NF-κB/AIM2 pathway in the ischemic cortex of CIRI rats

**FIGURE 6:**
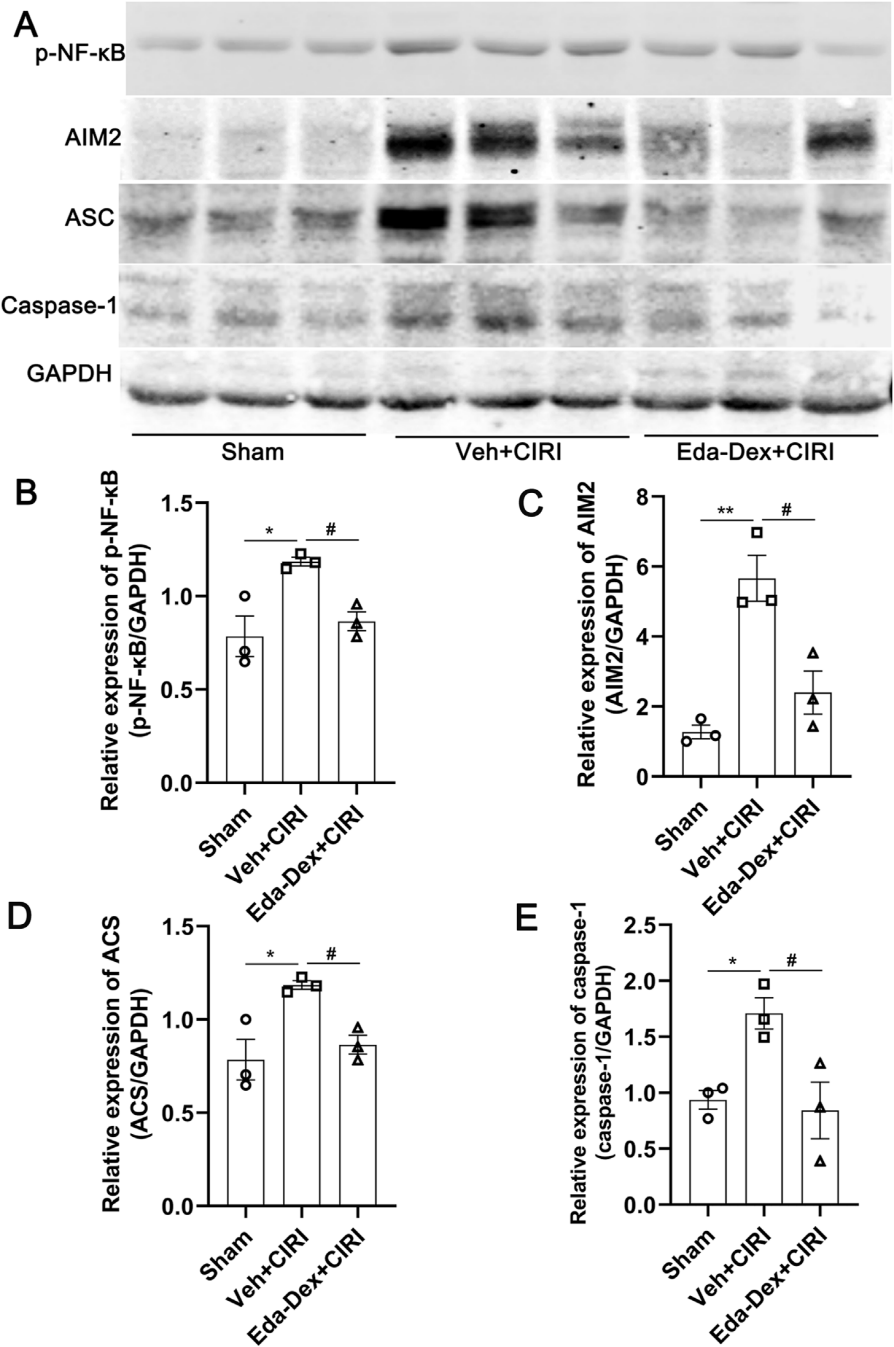
Influences of Eda-Dex on the NF-κB/AIM2 pathway. **(A)** Representative images of Western blotting and quantification of the expression levels of **(B)** p-NF-κB, **(C)** AIM2, **(D)** ASC, and **(E)** caspase-1. The expression levels of these proteins were significantly increased in response to CIRI, and were significantly decreased by the Eda-Dex treatment. The data are presented as the means±SEMs and were analyzed by one-way ANOVA followed by Tukey’s *post hoc* test (n = 3). *p < 0.05, and **p < 0.01 versus the sham group; ^#^ *p* < 0.05 versus the veh + CIRI group.

The expression of NF-κB/AIM2 signaling in the ischemic cortex was further analyzed. As shown in Figure 6, the levels of p-NF-κB, AIM2, ASC and caspase-1 were significantly greater in the Veh + CIRI group than in the sham group (*p =* 0.017; *p =* 0.0027; *p =* 0.017; *p =* 0.046), and Eda-Dex administration significantly inhibited these increases (*F* = 8.98, *p* = 0.043; *F* = 18.44, *p* = 0.011; *F* = 8.98, *p* = 0.043; *F* = 7.50, *p* = 0.029).

### 3.7 Eda-Dex ameliorated pathological injury and protected neurons in CIRI rats

**FIGURE 7:**
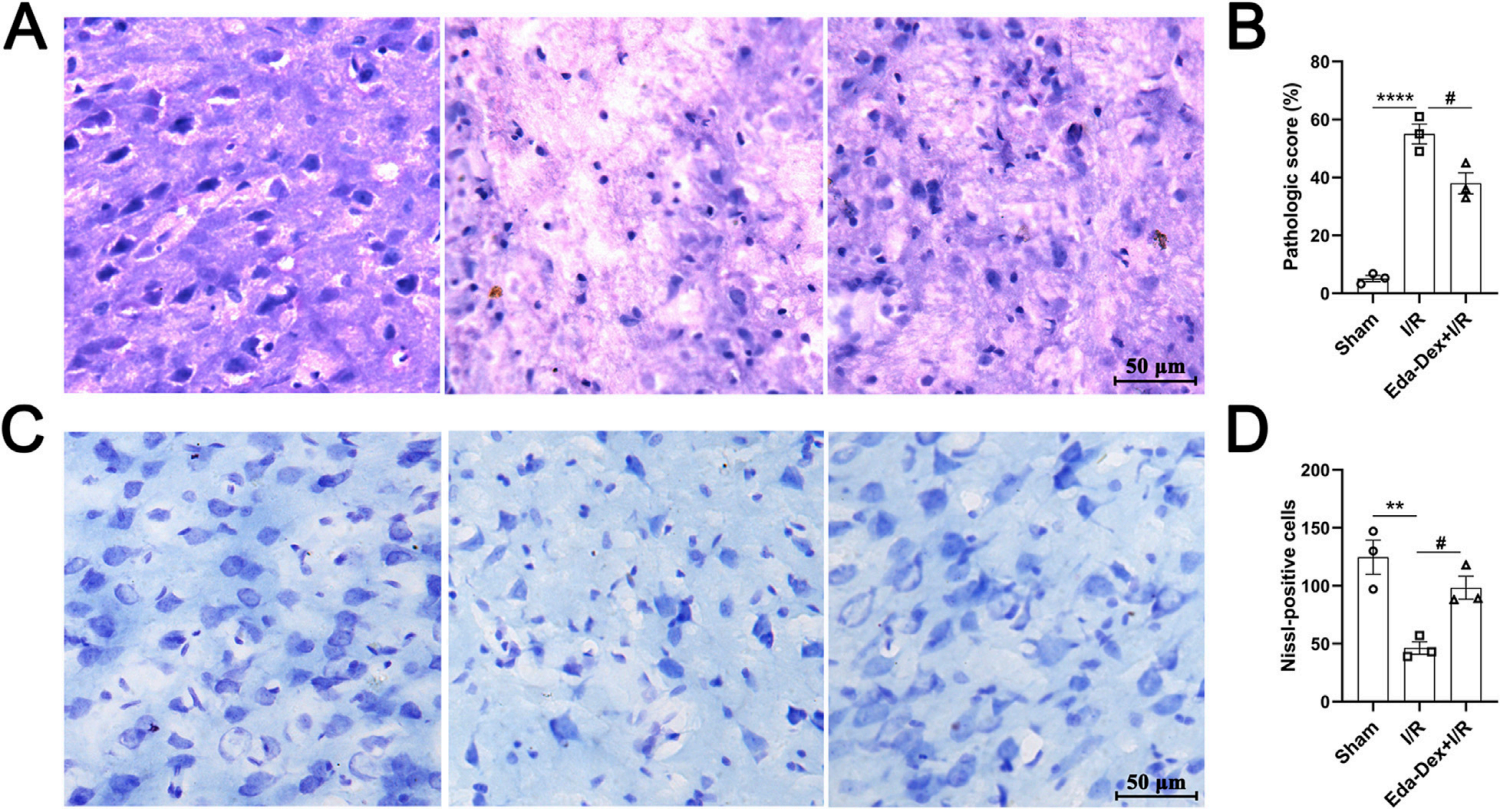
Influences of Eda-Dex on pathological changes in the infarct cortex. **(A)** Representative HE stained images of the right cerebral cortex of the animals in the three groups. **(B)** Pathologic score according to the HE images. Eda-Dex obviously restored neuronal structure and improved the histology of the damaged tissues. **(C)** Representative Nissl stained images of the right cerebral cortex in the three groups. **(D)** Number of Nissl bodies. Eda-Dex significantly increased the number of neurons in the infarct cortex. The data are presented as the means±SEMs and were analyzed by one-way ANOVA followed by Tukey’s *post hoc* test (n = 3). **p < 0.01, and ****p < 0.0001 versus the sham group; ^#^ *p* < 0.05 versus the veh + CIRI group. Scale bar = 50 μm.

The impact of Eda-Dex administration on pathological alterations in the infarcted cortex was assessed via HE and Nissl staining. As shown in Figure 7A, the nerve cells in the control animals appear neatly organized and structurally sound. In the animals in the CIRI group, neuronal structure is severely damaged, the nuclei of the cells are deformed and atrophied, the number of nerve cells is reduced, and the cells are loosely arranged. Compared with that in the CIRI group, neuronal structure was obviously restored, and the histology of the damaged tissues was dramatically improved by Eda-Dex treatment (*F* = 74.00, *p* = 0.015) (Figure 7B). Nissl staining revealed a significant loss of neurons in the infarct cortex of the CIRI group compared with the sham group (*p* < 0.01). Compared with CIRI, Eda-Dex treatment significantly increased the number of rescued neurons in the infarct cortex (*F* = 13.94, *p* = 0.032) (Figures 7C, D).

### 3.8 Eda-Dex suppressed the activation of astrocytes and microglia in CIRI rats

**FIGURE 8:**
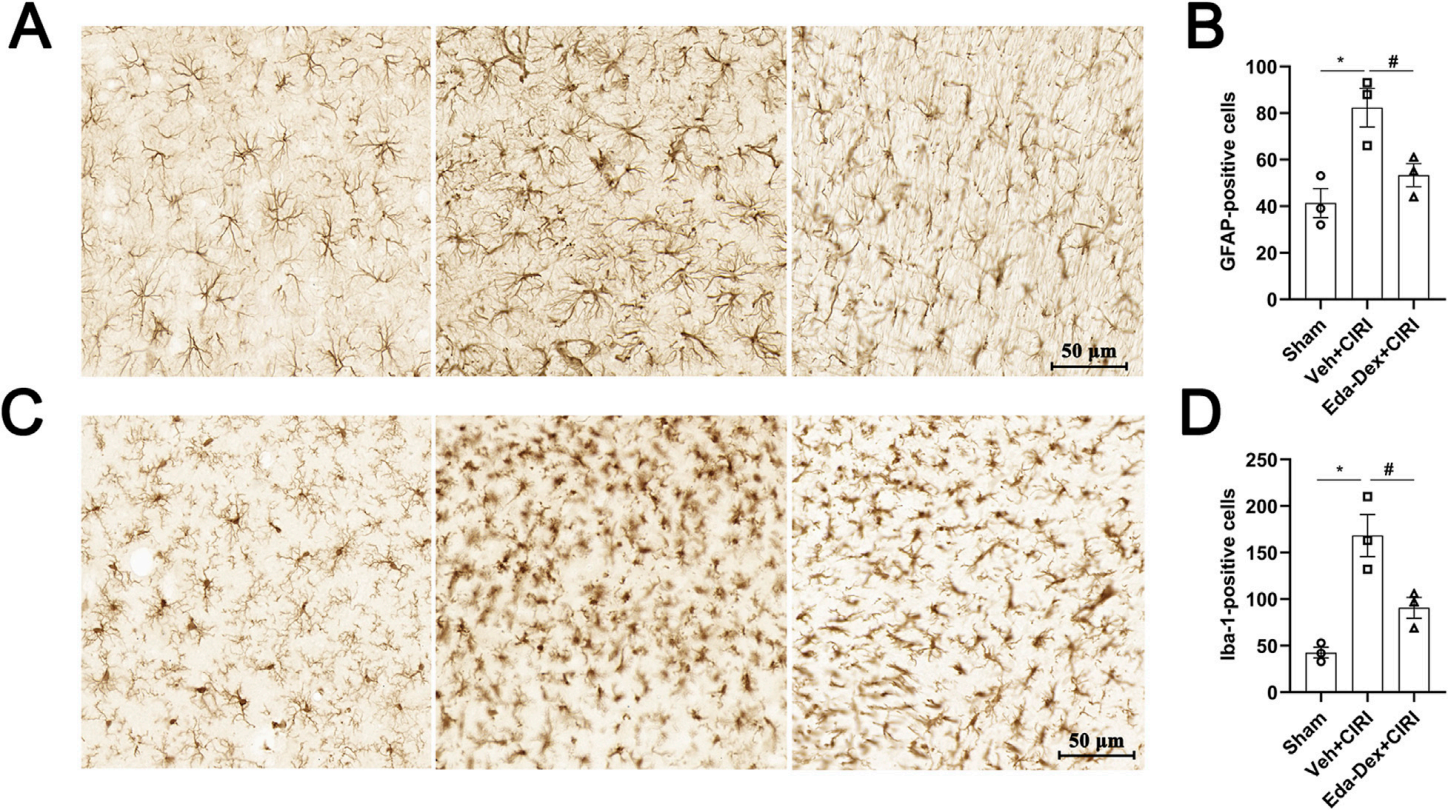
Effects of Eda-Dex on the activation of microglia and astrocytes. The quantity and morphology of microglia and astrocytes were analyzed by immunochemistry. Eda-Dex significantly decreased GFAP-positive cells **(A, B)** and Iba1-positive cells **(C, D)**, suggesting that Eda-Dex could protect microglia and astrocytes against overactivation in CIRI rats. The data are presented as the means±SEMs and were analyzed by one-way ANOVA followed by Tukey’s *post hoc* test (n = 3). *p < 0.05 versus the sham group; ^#^ *p* < 0.05 versus the veh + CIRI group. Scale bar = 50 μm.

The populations and morphological characteristics of astrocytes and microglia in the infarct cortex were evaluated by immunochemistry. In the sham group, the number and volume of astrocytes were small, whereas the number of astrocytes in the model group was significantly greater (*p* = 0.011), the cell volume was greater, and the cellular protrusions were more numerous and thicker. After Eda-Dex intervention, the number of astrocytes decreased significantly (*F* = 10.12, *p* = 0.049), the cell volume decreased, and the number of protrusions decreased (Figures 8A, B). The number of microglia in the sham group was small, and the microglia that were present had branched and elongated protrusions. The number of microglia in the Veh+CIRI group was significantly greater than that in the sham group (*p* = 0.0025); the microglia had more numerous and thicker protrusions, the connections among the protrusions were greater, and the volume of the microglia was greater. After Eda-Dex intervention, the number of microglia decreased significantly (*F* = 17.96, *p* = 0.024), and the protrusions became thinner (Figures 8C, D). The above data suggest that Eda-Dex effectively attenuates the overactivation of astrocytes and microglia in CIRI rats.

## 4 Discussion

Stroke patients commonly suffer from neurological dysfunction and poststroke cognitive impairment (PSCI), manifesting as depression, anxiety, and impaired learning and memory ability. Current therapies for neurological and cognitive dysfunction caused by stroke are controversial because of the complexity of stroke (Filler et al., 2024). Neuroprotectants that target multiple pathways have shown potential in stroke treatment. During ischemic stroke, an irreversible necrotic core and a penumbra surrounding the core are formed. Current findings suggest that the penumbra can be rescued by timely treatment with neuroprotective reagents (Belayev et al., 2018; Hui et al., 2024).

Both clinical and basic studies have demonstrated that Eda-Dex is an effective neuroprotective agent for ischemic stroke treatment. Multiple pathways, including inflammation, oxidative stress, and apoptosis, were involved in the protective effects of Eda-Dex (Chen Z. et al., 2024; Hu X et al., 2023). In consistent to these studies, our data obtained from animal experiments showed that Eda-Dex dramatically attenuated neurological deficits, alleviated brain damage and improved CBF in CIRI rats. Accordingly, pathological injury and neurons loss were obviously ameliorated by Eda-Dex. Overactivation of microglia and astrocytes after ischemic stroke is involved in the process of neuroinflammation (Orihuela et al., 2016). A recent study showed that Eda-Dex could promote the transformation of microglia and astrocytes from pro-inflammatory (M1/A1) phenotype to anti-inflammatory (M2/A2) phenotypes (Wang et al., 2024). According to our immunohistochemical results, the number of microglia and astrocytes increased dramatically, and their morphology changed obviously after CIRI, whereas Eda-Dex effectively prevented the excessive activation.

The influence of Eda-Dex on cognitive function after stroke was subsequently explored via behavioral tests. Although drugs such as cholinesterase inhibitors and memantine have shown beneficial effects in individuals with poststroke cognitive impairment, the effects of these drugs are still controversial (O'Sullivan et al., 2023). Eda-Dex has been confirmed to potentially improve cognitive function in various models of disease, including models of bilateral carotid artery stenosis, vascular dementia, and ischemic stroke (Li L. et al., 2023; Zhang J. et al., 2023; Zhang et al., 2023c). Previou studies have shown that anxiety is prevalent after stroke and is closely related to PSCI (Ruthmann et al., 2025; Liu et al., 2023; Tan et al., 2024). Therefore, the anxiety-like behavior in CIRI rats was analyzed by OFT and EPM test in our study. The results showed that the CIRI rats spent significantly less time in the central area in OFT and in the open arms in EPM compared with the normal rats, indicating that CIRI led to anxiety-like behavior. Eda-Dex greatly prolonged the time spent by rats in the central area during the OFT and the time spent in the open arms in the EPM test, suggesting that Eda-Dex administration can attenuate anxiety-like behaviors. It has been reported that animals that have experienced stroke exhibit obvious learning and memory deficits (Bu et al., 2019). The NOR test results obtained in our study revealed that CIRI rats presented obvious learning and memory deficits, whereas Eda-Dex administration effectively ameliorated learning and memory impairments in CIRI rats. The above data confirm that Eda-Dex potently improves neurological function, attenuates anxiety-like behavior and PSCI in CIRI rats.

Eda-Dex combines the activities of edaravone and (+)-borneol and has both antioxidative stress and anti-inflammatory functions (Shi et al., 2022). NRF2 is recognized as a key oxidative stress regulator and moderate expression of NRF2 is conducive to reducing brain injury and promoting long-term recovery from brain injury after cerebral ischemia (Liu et al., 2019). Studies revealed that Eda-Dex potently activates NRF2 by inhibiting Keap1 (Zhang et al., 2023c) or activating MKP-1 (Zhang et al., 2022) and effectively attenuates cerebral ischemic injury. In the meanwhile, the downstream molecules of NRF2, such as HO-1, SLC7A11, and GPX4, by which Eda-Dex exserts anti-oxidative stress function, have been investigated is ischemic stroke (Xu et al., 2022; Xiao et al., 2024). In our study, the transcripts of NRF2, including HO-1, NQO1, and SLC7A11, were systematically analyzed. HO-1 has multiple activities and plays a protective role in brain tissue (Dang et al., 2022; Zhang Y. et al., 2024). NQO1 uses NAPDH as an electron donor to reduce quinones and thus prevents the formation of oxygen free radicals during the oxygenation reaction (Yang et al., 2024). Notably, recent studies suggest that genes related to ferroptosis, such as SLC7A11, are also transcriptional targets of NRF2 (Dodson et al., 2019; Chen et al., 2023; Li et al., 2019; Li et al., 2022; Xiang et al., 2024). Our results revealed that Eda-Dex significantly activated NRF2 and upregulated the downstream proteins. In addition, we found that Eda-Dex potently decreased ROS and MDA and elevated T-AOC in CIRI rats. The above data suggested that Eda-Dex alleviates oxidative stress by activating the NRF2/ARE pathway. Although the expression of SLC7A11, were found to be upregulated by Eda-Dex, the mechanism of Eda-Dex in ferroptosis in ischemic stroke is still to be investigated.

Neuroinflammation causes a secondary wave of damage to the brain following ischemic stroke. The NF-κB pathway has been suggested to be involved in inflammatory response during ischemic stroke (Wróbel-Biedrawa and Podolak, 2024; Chen et al., 2019). NF-κB plays a key role in the initiation of the inflammasome, which has been shown to play an important role in the pathogenesis of ischemic stroke (Long et al., 2023; Olsen et al., 2021).

The AIM2 inflammasome, which is composed of AIM2, ASC and caspase-1, is one of the most widely studied inflammasome. Clinical studies have shown a significant increase in the number of penumbral AIM2^+^/CD68^+^ cells in stroke patients (Matsuyama et al., 2020). In animal models of stroke and cell models of oxygen‒glucose deprivation/reperfusion, the AIM2 inflammasome is significantly activated, and this is accompanied by neuronal pyroptosis (Liang et al., 2020). By inhibiting microglial pyroptosis through regulation of the AIM2, the neuroinflammatory response and ischemic brain injury can be alleviated, and nerve function can be improved (Li et al., 2020). AIM2-induced cell death is the main cause of neuronal death and that knockout of AIM2 significantly improved spatial learning and memory capability in mice (Kim et al., 2020). Recent studies suggested that inhibition of NF-κB/AIM2 signaling pathway contributed to attenuating neuroinflammation and improving neurological function in intracerebral hemorrhage (Li X. et al., 2023) and ischemic stroke (Zhao et al., 2024).

The inhibitory effect of Eda-Dex on NF-κB in a previous study have been confirmed in several studies (Huang et al., 2024; Chen W. et al., 2024). However, the regulatory effect of Eda-Dex on NF-κB/AIM2 pathway is still unknown. Our results showed that Eda-Dex significantly downregulated the protein levels of p-NF-κB, AIM2, ASC, and caspase-1 and suppressed the production of inflammatory cytokines. The data suggests that Eda-Dex may attenuate neuroinflammation by targeting the NF-κB/AIM2 pathway.

In this study, the work presented here analyzed the protective effect of Eda-Dex on neurological function in stroke through synergistic anti-inflammatory and antioxidant effects, and explored the underling mechanisms in CIRI rats. The data showed that Eda-Dex potently attenuates oxidative stress and inflammation in the ischemic brain and that the NRF/ARE and NF-κB/AIM2 pathways may be involved in these processes. This study provides new experimental evidence for the mechanism of Eda-Dex in neurological protection for CIRI, and provides new theoretical support for the clinical usage of Eda-Dex in ischemic stroke. However, this study has certain limitations. First, given the complexity of stroke pathogenesis, the relevance of crosstalk between the NRF/ARE pathway and the NF-κB/AIM2 pathway during PSCI is still unclear. Second, although this study confirmed that Eda-Dex has regulatory effects on both of these pathways, the specific effects of Eda-Dex on its target molecules have not yet been clarified. Ultimately, this research relies on animal models, and additional clinical studies are necessary to validate the findings.

## 5 Conclusion

**FIGURE 9:**
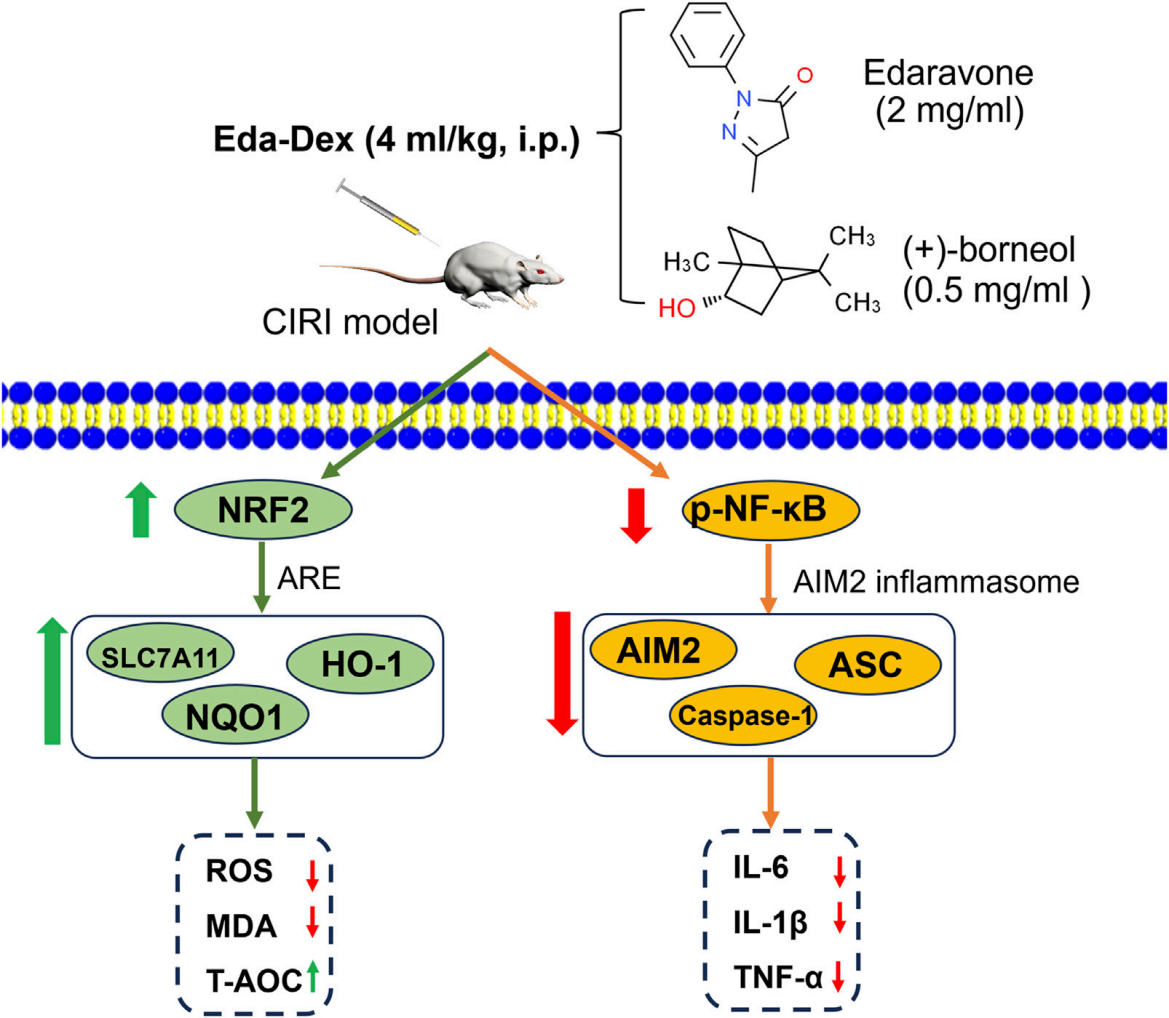
Mechanism underlying the anti-inflammatory and anti-oxidative stress effects of Eda-Dex in the CIRI model.

Eda-Dex administration potently attenuated cerebral damage, protected neurological function, attenuated anxiety-like behavior and improved cognitive function by synergistically inhibiting oxidative stress and inflammation through targeting of the NRF2/ARE and NF-κB/AIM2 pathways in CIRI rats (Figure 9). Eda-Dex is a promising neuroprotectant for treating ischemic stroke.

## Data Availability

The original contributions presented in the study are included in the article/supplementary material, further inquiries can be directed to the corresponding authors.

## References

[B1] Bao M. H. Li J. M. Zhou Q. L. Li G. Y. Zeng J. Zhao J. (2016). Effects of miR 590 on oxLDL induced endothelial cell apoptosis: roles of p53 and NF κB. Mol. Med. Rep. 13 (1), 867–873. 10.3892/mmr.2015.4606 26648441

[B2] Belayev L. Hong S. H. Menghani H. Marcell S. J. Obenaus A. Freitas R. S. (2018). Docosanoids promote neurogenesis and angiogenesis, blood-brain barrier integrity, penumbra protection, and neurobehavioral recovery after experimental ischemic stroke. Mol. Neurobiol. 55 (8), 7090–7106. 10.1007/s12035-018-1136-3 29858774 PMC6054805

[B3] Bu F. Min J. W. Munshi Y. Lai Y. J. Qi L. Urayama A. (2019). Activation of endothelial ras-related C3 botulinum toxin substrate 1 (Rac1) improves post-stroke recovery and angiogenesis via activating Pak1 in mice. Exp. Neurol. 322, 113059. 10.1016/j.expneurol.2019.113059 31499064 PMC6864282

[B4] Chen J. Li X. Liu H. Zhong D. Yin K. Li Y. (2023). Bone marrow stromal cell-derived exosomal circular RNA improves diabetic foot ulcer wound healing by activating the nuclear factor erythroid 2-related factor 2 pathway and inhibiting ferroptosis. Diabet. Med. 40 (7), e15031. 10.1111/dme.15031 36537855

[B5] Chen S. Zhao Y. Shen F. Long D. Yu T. Lin X. (2019). Introduction of exogenous wild-type p53 mediates the regulation of oncoprotein 18/stathmin signaling via nuclear factor-κB in non-small cell lung cancer NCI-H1299 cells. Oncol. Rep. 41 (3), 2051–2059. 10.3892/or.2019.6964 30628717

[B6] Chen W. Zhang H. Li Z. Deng Q. Wang M. Chen Y. (2024a). Effects of edaravone dexborneol on functional outcome and inflammatory response in patients with acute ischemic stroke. BMC Neurol. 24 (1), 209. 10.1186/s12883-024-03712-1 38902691 PMC11188235

[B7] Chen Z. Li T. Tang H. B. Lu Z. W. Chen Z. Y. Zhao Z. H. (2024b). Edaravone Dexborneol provides neuroprotective effect by inhibiting neurotoxic activation of astrocytes through inhibiting NF-κB signaling in cortical ischemia. Brain Res. Bull. 218, 111097. 10.1016/j.brainresbull.2024.111097 39395778

[B8] Dang R. Wang M. Li X. Wang H. Liu L. Wu Q. (2022). Edaravone ameliorates depressive and anxiety-like behaviors via Sirt1/Nrf2/HO-1/Gpx4 pathway. J. Neuroinflammation 19 (1), 41. 10.1186/s12974-022-02400-6 35130906 PMC8822843

[B9] Dodson M. Castro-Portuguez R. Zhang D. D. (2019). NRF2 plays a critical role in mitigating lipid peroxidation and ferroptosis. Redox Biol. 23, 101107. 10.1016/j.redox.2019.101107 30692038 PMC6859567

[B10] Dong H. Qiang Z. Chai D. Peng J. Xia Y. Hu R. (2020). Nrf2 inhibits ferroptosis and protects against acute lung injury due to intestinal ischemia reperfusion via regulating SLC7A11 and HO-1. Aging (Albany NY) 12 (13), 12943–12959. 10.18632/aging.103378 32601262 PMC7377827

[B11] Dong T. Chen N. Ma X. Wang J. Wen J. Xie Q. (2018). The protective roles of L-borneolum, D-borneolum and synthetic borneol in cerebral ischaemia via modulation of the neurovascular unit. Biomed. Pharmacother. 102, 874–883. 10.1016/j.biopha.2018.03.087 29728011

[B12] Filler J. Georgakis M. K. Dichgans M. (2024). Risk factors for cognitive impairment and dementia after stroke: a systematic review and meta-analysis. Lancet Healthy Longev. 5 (1), e31–e44. 10.1016/S2666-7568(23)00217-9 38101426

[B13] Fu R. Zhao L. Guo Y. Qin X. Xu W. Cheng X. (2024a). AIM2 inflammasome: a potential therapeutic target in ischemic stroke. Clin. Immunol. 259, 109881. 10.1016/j.clim.2023.109881 38142900

[B14] Fu Y. Wang A. Tang R. Li S. Tian X. Xia X. (2024b). Sublingual edaravone dexborneol for the treatment of acute ischemic stroke: the TASTE-SL randomized clinical trial. JAMA Neurol. 81 (4), 319–326. 10.1001/jamaneurol.2023.5716 38372981 PMC10877503

[B15] Herpich F. Rincon F. (2020). Management of acute ischemic stroke. Crit. Care. Med. 48 (11), 1654–1663. 10.1097/CCM.0000000000004597 32947473 PMC7540624

[B16] Hilkens N. A. Casolla B. Leung T. W. de Leeuw F. E. (2024). Stroke. Lancet 403 (10446), 2820–2836. 10.1016/S0140-6736(24)00642-1 38759664

[B17] Hu X. Qian Z. Chen J. Chen M. Zhong W. Shen C. (2023). Effects of edaravone dexborneol on neurological function and serum inflammatory factor levels in patients with acute anterior circulation large vessel occlusion stroke. Transl. Neurosci. 14 (1), 20220312. 10.1515/tnsci-2022-0312 37854582 PMC10579784

[B18] Hua K. Sheng X. Li T. T. Wang L. N. Zhang Y. H. Huang Z. J. (2015). The edaravone and 3-n-butylphthalide ring-opening derivative 10b effectively attenuates cerebral ischemia injury in rats. Acta. Pharmacol. Sin. 36 (8), 917–927. 10.1038/aps.2015.31 26073328 PMC4564877

[B19] Huang J. Hu X. Li J. Gong D. (2024). Edaravone dexborneol promotes M2 microglia polarization against lipopolysaccharide-induced inflammation via suppressing TLR4/MyD88/NF-κB pathway. Naunyn Schmiedeb. Arch. Pharmacol. 397 (9), 6647–6659. 10.1007/s00210-024-03045-3 38489082

[B20] Hugues N. Pellegrino C. Rivera C. Berton E. Pin-Barre C. Laurin J. (2021). Is high-intensity interval training suitable to promote neuroplasticity and cognitive functions after stroke? Int. J. Mol. Sci. 22 (6), 3003. 10.3390/ijms22063003 33809413 PMC7998434

[B21] Hui Z. LaiFa W. XueQin W. Ling D. BinSheng H. Li J. M. (2024). Mechanisms and therapeutic potential of chinonin in nervous system diseases. J. Asian Nat. Prod. Res. 26 (12), 1405–1420. 10.1080/10286020.2024.2371040 38975978

[B22] Ishihara H. Suzuki M. (2016). Japanese guidelines for the management of stroke 2015: overview of the chapter on subarachnoid hemorrhage. Nihon. Rinsho. 74 (4), 677–680. 27333759

[B23] Jaiswal S. Hockenbury N. Pan H. Knutsen A. Dardzinski B. J. Byrnes K. R. (2019). Alteration of FDG uptake by performing novel object recognition task in a rat model of traumatic brain injury. Neuroimage 188, 419–426. 10.1016/j.neuroimage.2018.12.033 30576849 PMC6401244

[B24] Kim H. Seo J. S. Lee S. Y. Ha K. T. Choi B. T. Shin Y. I. (2020). AIM2 inflammasome contributes to brain injury and chronic post-stroke cognitive impairment in mice. Brain Behav. Immun. 87, 765–776. 10.1016/j.bbi.2020.03.011 32201254

[B25] Lee T. H. Yeh J. C. Tsai C. H. Yang J. T. Lou S. L. Seak C. J. (2017). Improved thrombolytic effect with focused ultrasound and neuroprotective agent against acute carotid artery thrombosis in rat. Sci. Rep. 7 (1), 1638. 10.1038/s41598-017-01769-2 28487554 PMC5431649

[B26] Li F. Li D. Liu H. Cao B. B. Jiang F. Chen D. N. (2019). RNF216 regulates the migration of immortalized GnRH neurons by suppressing beclin1-mediated autophagy. Front. Endocrinol. (Lausanne). 10, 12. 10.3389/fendo.2019.00012 30733708 PMC6354547

[B27] Li L. He G. Shi M. Zhu J. Cheng Y. Chen Y. (2023a). Edaravone dexborneol ameliorates cognitive impairment by regulating the NF-κB pathway through AHR and promoting microglial polarization towards the M2 phenotype in mice with bilateral carotid artery stenosis (BCAS). Eur. J. Pharmacol. 957, 176036. 10.1016/j.ejphar.2023.176036 37673366

[B28] Li L. Wang S. Zhou W. (2022). Balance cell apoptosis and pyroptosis of caspase-3-activating chemotherapy for better antitumor therapy. Cancers (Basel) 15 (1), 26. 10.3390/cancers15010026 36612023 PMC9817729

[B29] Li Q. Cao Y. Dang C. Han B. Han R. Ma H. (2020). Inhibition of double-strand DNA-sensing cGAS ameliorates brain injury after ischemic stroke. EMBO Mol. Med. 12 (4), e11002. 10.15252/emmm.201911002 32239625 PMC7136961

[B30] Li X. Zhang H. Zheng W. Sun J. Wang L. He Z. (2023b). Ozanimod-dependent activation of SIRT3/NF-κB/AIM2 pathway attenuates secondary injury after intracerebral hemorrhage. Mol. Neurobiol. 60 (3), 1117–1131. 10.1007/s12035-022-03137-2 36417102

[B31] Liang D. Guan Q. Huang M. He Y. Ou Y. Chen M. (2023). Changing trends of disease burden of stroke from 1990 to 2019 and its predictions among the Chinese population. Front. Neurol. 14, 1255524. 10.3389/fneur.2023.1255524 37869143 PMC10588696

[B32] Liang J. Wang Q. Li J. Q. Guo T. Yu D. (2020). Long non-coding RNA MEG3 promotes cerebral ischemia-reperfusion injury through increasing pyroptosis by targeting miR-485/AIM2 axis. Exp. Neurol. 325, 113139. 10.1016/j.expneurol.2019.113139 31794744

[B33] Liu H. Kong L. Sun Q. Ma X. (2023). The effects of mindfulness-based interventions on nurses' anxiety and depression: a meta-analysis. Nurs. Open 10 (6), 3622–3634. 10.1002/nop2.1610 36694384 PMC10170883

[B34] Liu L. Locascio L. M. Doré S. (2019). Critical role of Nrf2 in experimental ischemic stroke. Front. Pharmacol. 10, 153. 10.3389/fphar.2019.00153 30890934 PMC6411824

[B35] Long J. X. Tian M. Z. Chen X. Y. Yu H. H. Ding H. Liu F. (2023). The role of NLRP3 inflammasome-mediated pyroptosis in ischemic stroke and the intervention of traditional Chinese medicine. Front. Pharmacol. 14, 1151196. 10.3389/fphar.2023.1151196 37153784 PMC10160381

[B36] Longa E. Z. Weinstein P. R. Carlson S. Cummins R. (1989). Reversible middle cerebral artery occlusion without craniectomy in rats. Stroke 20 (1), 84–91. 10.1161/01.str.20.1.84 2643202

[B37] Lou Z. Wang A. P. Duan X. M. Hu G. H. Zuo M. L. Yang Z. B. (2018). Role of ALK5/SMAD2/3 signaling in the regulation of NOX expression in cerebral ischemia/reperfusion injury. Exp. Ther. Med. 16 (3), 1671–1678. 10.3892/etm.2018.6377 30186386 PMC6122315

[B38] Luo G. Zhou Z. Huang C. Zhang P. Sun N. Chen W. (2023). Itaconic acid induces angiogenesis and suppresses apoptosis via Nrf2/autophagy to prolong the survival of multi-territory perforator flaps. Heliyon 9 (7), e17909. 10.1016/j.heliyon.2023.e17909 37456049 PMC10345368

[B39] Matsuyama H. Shindo A. Shimada T. Yata K. Wakita H. Takahashi R. (2020). Chronic cerebral hypoperfusion activates AIM2 and NLRP3 inflammasome. Brain Res. 1736, 146779. 10.1016/j.brainres.2020.146779 32171704

[B40] Olsen M. B. Gregersen I. Sandanger Ø. Yang K. Sokolova M. Halvorsen B. E. (2021). Targeting the inflammasome in cardiovascular disease. JACC Basic Transl. Sci. 7 (1), 84–98. 10.1016/j.jacbts.2021.08.006 35128212 PMC8807732

[B41] Orihuela R. McPherson C. A. Harry G. J. (2016). Microglial M1/M2 polarization and metabolic states. Br. J. Pharmacol. 173 (4), 649–665. 10.1111/bph.13139 25800044 PMC4742299

[B42] O'Sullivan M. J. Li X. Galligan D. Pendlebury S. T. (2023). Cognitive recovery after stroke: memory. Stroke 54 (1), 44–54. 10.1161/STROKEAHA.122.041497 36542075

[B43] Ruthmann F. Lo J. W. Mendyk-Bordet A. M. Allart E. Köhler S. Klimkowicz-Mrowiec A. (2025). Prevalence of poststroke anxiety and its associations with global cognitive impairment: an individual participant data analysis. J. Affect. Disord. 369, 1136–1144. 10.1016/j.jad.2024.10.099 39447966

[B44] Shah Z. A. Namiranian K. Klaus J. Kibler K. Doré S. (2006). Use of an optimized transient occlusion of the middle cerebral artery protocol for the mouse stroke model. J. Stroke Cerebrovasc. Dis. 15 (4), 133–138. 10.1016/j.jstrokecerebrovasdis.2006.04.002 17904065

[B45] Shi F. He Z. Wang L. Su H. Han S. (2022). Cost-effectiveness of edaravone dexborneol versus edaravone for the treatment of acute ischemic stroke in China: based on the TASTE study. Front. Pharmacol. 13, 938239. 10.3389/fphar.2022.938239 36330098 PMC9622952

[B46] Tan X. He Y. Ning N. Peng J. Wiley J. Fan F. (2024). Shared decision-making in the treatment of adolescents diagnosed with depression: a cross-sectional survey of mental health professionals in China. J. Psychiatr. Ment. Health Nurs. 31 (3), 340–351. 10.1111/jpm.12990 37882490

[B47] Wang D. Wang Y. Shi J. Jiang W. Huang W. Chen K. (2024). Edaravone dexborneol alleviates ischemic injury and neuroinflammation by modulating microglial and astrocyte polarization while inhibiting leukocyte infiltration. Int. Immunopharmacol. 130, 111700. 10.1016/j.intimp.2024.111700 38382262

[B48] Wang X. An F. Wang S. An Z. Wang S. (2017). Orientin attenuates cerebral ischemia/reperfusion injury in rat model through the AQP-4 and TLR4/NF-κB/TNF-α signaling pathway. J. Stroke Cerebrovasc. Dis. 26 (10), 2199–2214. 10.1016/j.jstrokecerebrovasdis.2017.05.002 28645524

[B49] Wróbel-Biedrawa D. Podolak I. (2024). Anti-neuroinflammatory effects of adaptogens: a mini-review. Molecules 29 (4), 866. 10.3390/molecules29040866 38398618 PMC10891670

[B50] Wu H. Y. Tang Y. Gao L. Y. Sun W. X. Hua Y. Yang S. B. (2014). The synergetic effect of edaravone and borneol in the rat model of ischemic stroke. Eur. J. Pharmacol. 740, 522–531. 10.1016/j.ejphar.2014.06.035 24975100

[B51] Xiang Y. Song X. Long D. (2024). Ferroptosis regulation through Nrf2 and implications for neurodegenerative diseases. Arch. Toxicol. 98 (3), 579–615. 10.1007/s00204-023-03660-8 38265475 PMC10861688

[B52] Xiao P. Huang H. Zhao H. Liu R. Sun Z. Liu Y. (2024). Edaravone dexborneol protects against cerebral ischemia/reperfusion-induced blood-brain barrier damage by inhibiting ferroptosis via activation of nrf-2/HO-1/GPX4 signaling. Free Radic. Biol. Med. 217, 116–125. 10.1016/j.freeradbiomed.2024.03.019 38548187

[B53] Xu J. Wang A. Meng X. Yalkun G. Xu A. Gao Z. (2021). Edaravone dexborneol versus edaravone alone for the treatment of acute ischemic stroke: a phase iii, randomized, double-blind, comparative trial. Stroke 52 (3), 772–780. 10.1161/STROKEAHA.120.031197 33588596

[B54] Xu L. Gao Y. Hu M. Dong Y. Xu J. Zhang J. (2022). Edaravone dexborneol protects cerebral ischemia reperfusion injury through activating Nrf2/HO-1 signaling pathway in mice. Fundam. Clin. Pharmacol. 36 (5), 790–800. 10.1111/fcp.12782 35470467 PMC9545784

[B55] Yang K. Yu X. Guo Z. Fang Z. Zhang H. Zhang W. (2024). PIM1 alleviated liver oxidative stress and NAFLD by regulating the NRF2/HO-1/NQO1 pathway. Life Sci. 349, 122714. 10.1016/j.lfs.2024.122714 38735366

[B56] Yao Q. Y. Fu M. L. Zhao Q. Zheng X. M. Tang K. Cao L. M. (2023). Image-based visualization of stents in mechanical thrombectomy for acute ischemic stroke: preliminary findings from a series of cases. World J. Clin. Cases 11 (21), 5047–5055. 10.12998/wjcc.v11.i21.5047 37583850 PMC10424012

[B57] Yi J. Li L. Yin Z. J. Quan Y. Y. Tan R. R. Chen S. L. (2023). Polypeptide from moschus suppresses lipopolysaccharide-induced inflammation by inhibiting NF-κB-ROS/NLRP3 Pathway. Chin. J. Integr. Med. 29 (10), 895–904. 10.1007/s11655-023-3598-z 37542626

[B58] Zhang H. Wang L. Wang X. Deng L. He B. Yi X. (2024a). Mangiferin alleviated poststroke cognitive impairment by modulating lipid metabolism in cerebral ischemia/reperfusion rats. Eur. J. Pharmacol. 977, 176724. 10.1016/j.ejphar.2024.176724 38851559

[B59] Zhang H. Wang L. Yang Y. Cai C. Wang X. Deng L. (2023a). DL-3-n-butylphthalide (NBP) alleviates poststroke cognitive impairment (PSCI) by suppressing neuroinflammation and oxidative stress. Front. Pharmacol. 13, 987293. 10.3389/fphar.2022.987293 36712684 PMC9878832

[B60] Zhang H. Wang L. Zhu B. Yang Y. Cai C. Wang X. (2023c). A comparative study of the neuroprotective effects of dl-3-n-butylphthalide and edaravone dexborneol on cerebral ischemic stroke rats. Eur. J. Pharmacol. 951, 175801. 10.1016/j.ejphar.2023.175801 37207969

[B61] Zhang J. Xiao Y. Liu H. Xu L. Guo X. Gao Y. (2023b). Edaravone dexborneol alleviates neuroinflammation by reducing neuroglial cell proliferation and suppresses neuronal apoptosis/autophagy in vascular dementia rats. Neurochem. Res. 48 (10), 3113–3128. 10.1007/s11064-023-03973-1 37338792

[B62] Zhang W. Yang H. Gao M. Zhang H. Shi L. Yu X. (2022). Edaravone dexborneol alleviates cerebral ischemic injury via MKP-1-mediated Inhibition of MAPKs and activation of Nrf2. Biomed. Res. Int. 2022, 4013707. 10.1155/2022/4013707 36110124 PMC9470337

[B63] Zhang Y. Zhang P. Zhang X. Liu Y. (2024b). HH-A, a modified honokiol, protects against cerebral ischemia/reperfusion induced brain injury in rodent via Nrf2/HO-1 signaling pathways. Naunyn. Schmiedeb. Arch. Pharmacol. 397 (5), 3389–3402. 10.1007/s00210-023-02816-8 37955691

[B64] Zhao C. Fu X. Yang Z. Zhang Q. Zhao Y. (2024). ATP-sensitive potassium channel opener, Nicorandil, inhibits NF-κB/AIM2/GSDMD pathway activation to protect against neuroinflammation in ischemic stroke. Neurochem. Int. 179, 105810. 10.1016/j.neuint.2024.105810 39069080

